# The status of infection prevention and control structures in Eastern China based on the IPCAF tool of the World Health Organization

**DOI:** 10.1186/s13756-022-01087-x

**Published:** 2022-03-09

**Authors:** Kaiwen Ni, Dingping Jin, Zhe Wu, Liyuan Sun, Qun Lu

**Affiliations:** 1 Department of Infection Control, Second Affiliated Hospital of Zhejiang University School of Medicine, Hangzhou, Zhejiang 310009 China; 2 Zhejiang Nosocomial Infection Control and Quality Improvement Center, Hangzhou, Zhejiang 310009 China

**Keywords:** Healthcare-associated infection, Infection prevention and control, Survey, WHO, Eastern China

## Abstract

**Background:**

The burden of healthcare-associated infections (HAIs) and the spread of antimicrobial resistance can be potentially preventable through comprehensive infection prevention and control (IPC) programs. However, information on the current state of IPC implementation is rare in China.

**Methods:**

A cross-sectional study was conducted in Zhejiang province, China, from April to June 2021. The Zhejiang nosocomial infection control and quality improvement center (NICQI) cooperated with 11 municipal NICQI centers to introduce the purpose of this study and invite all licensed secondary and tertiary hospitals in Zhejiang province through WeChat group. The questionnaire had three sections, including information about participating hospitals, demographic information about IPs, and the Chinese version of the Infection Prevention and Control Assessment Framework that covered eight core components (CC).

**Results:**

Of the 382 hospitals invited, 222 (58.1% response rate) accepted and completed the online questionnaire. The overall median score of the participating hospitals was 682 (630–723), which corresponded to an advanced level of IPC. There was a significant difference in scores between hospitals types (*P* < 0.001). Profound differences were revealed regarding the scores of the individual components, with CC2 (IPC guidelines) and CC6 (Monitoring/audit of IPC practices and feedback) having the highest (100) and lowest (65) median scores, respectively. Only 23 (10.4%) hospitals reported assessing facility safety culture.

**Conclusions:**

IPC structures are at a relatively high level in acute care hospitals in Eastern China. The identified potential areas for improvement were similar to those identified in developed countries, particularly regarding multimodal strategies for implementation and safety culture construction. Meanwhile, the Chinese government should pay more attention to IPC resources and practices among secondary care hospitals.

**Supplementary Information:**

The online version contains supplementary material available at 10.1186/s13756-022-01087-x.

## Background

Healthcare-associated infections (HAIs) are considered a global public health problem that can result in prolonged hospital stays, increased antimicrobial resistance, and reduced quality of life of patients [1, 2]. According to a World Health Organization (WHO) report, the prevalence of HAIs was 3.5%-12% in developed countries and 5.7%-19.1% in low- and middle-income countries [3]. A recent meta-analysis study reported that the prevalence of HAIs in China was 3.12% [4]. Infection prevention and control (IPC) reduces the burden of HAIs and the spread of antimicrobial resistance in acute care hospitals [5–7]. The importance of IPC has been amplified by the coronavirus disease 2019 (COVID-19) pandemic. However, determining ways to assess IPC resources and practices at the national or facility level remains a challenge.

In 2018, the National Health Commission of the People’s Republic of China published the “Accreditation regulation of control and prevention of healthcare associated infection in hospital”, which specifies basic principles, evaluation content and IPC requirements [8]. To support countries in their efforts to improve IPC practices, WHO released the IPC Assessment Framework (IPCAF), which is a structured questionnaire with an associated scoring system [9, 10]. The IPCAF contains eight core components (CC), which include IPC programme (CC1), IPC guidelines (CC2), IPC education and training (CC3), HAI surveillance (CC4), multimodal strategies for implementation of IPC interventions (CC5), monitoring/audit of IPC practices and feedback (CC6), workload, staffing and bed occupancy (CC7), built environment, materials and equipment for IPC at the facility level (CC8). Measurement is critical in the process of improving healthcare quality. IPCAF was developed to support the “baseline assessment” and “assessing impact” steps of WHO proposed IPC facility programmes. The baseline assessment is concerned with understanding the current IPC situation to guide action planning for improvement. The assessment impact is concerned with evaluating the effectiveness of activities undertaken in the context of the action plan. In recent years, IPCAF has been used to identify the strengths and weaknesses of IPC resources and practices in developed countries, demonstrating potentials for improvement, particularly in the implementation of multimodal strategies, IPC education and staffing [11, 12].

Infection preventionists (IPs) are responsible for education, surveillance, outbreak investigation, and implementation of IPC. IPs with diverse professional backgrounds have a positive impact on infection control departments, facilitating the implementation of innovative practices and therefore improving patient outcomes [13, 14]. However, information on the current state of IPC implementation is rare, especially during the COVID-19 pandemic in China. Thus, the purpose of this study was to evaluate staffing level, IPC resources, and practices in secondary and tertiary care hospitals.

## Methods

### Study design

This cross-sectional study was conducted in Zhejiang province, China, from April to June 2021. There are 11 cities and 382 secondary and tertiary care hospitals licensed by Health Commission of Zhejiang province. In 1994, a provincial-level center for nosocomial infection control and quality improvement (NICQI) was established in Zhejiang province, and then each city established its own NICQI center in the subsequent 20 years to address topics of annual interest regarding IPC programmes and continuing education. This survey was conducted by the provincial NICQI center in collaboration with 11 municipal NICQI centers, who introduced the purpose of this study and invited all licensed secondary and tertiary hospitals through WeChat groups. The study protocol was approved by the Ethic Committee of the Second Affiliated Hospital of Zhejiang University School of Medicine (No. 2021–0693).

### Measurement

The questionnaire had three sections. The first section handled hospital information, such as hospital type (secondary or tertiary care), hospital size (number of hospital beds), region (where the hospital is located), independent infection control department (yes or not), and establishment date of department. The second section was used to collect demographic information about IPs in the infection control department, including age, gender, background, education level, and years of service in the infection control department. The third section involved the Chinese version of the IPCAF that covered the eight CCs. A score was assigned to each answer to a question. The maximum score per CC was 100 and the highest possible overall score was 800. An IPC level was allocated to the hospitals based on the final score. A score of 0 to 200 was considered inadequate, 201–400 basic, 401–600 intermediate, and 601–800 advanced.

The provincial NICQI center translated the IPCAF into Chinese. A pilot survey was conducted in five potential participating hospitals in order to qualitatively identify question ambiguities and misunderstandings caused by translation. Along with the questionnaire, a guidance document, including study objectives, notes for filling out and terminology explanation, was also sent to all participants through WeChat groups in this study. Participants were informed that the survey was entirely voluntary and that the results were not associated with HAIs surveillance or hospital accreditation. The online questionnaire was completed by a full-time IP from the infection control department or in the absence of a full-time staff, by the relevant professional in charge of IPC activities. This online questionnaire was designed as all mandatory questions and must be completed before submission. Any queries were addressed via telephone or in the WeChat groups.

### Statistical analysis

All the data were analyzed using SPSS version 26 and STATA version 15. All results of the quantitative variables were presented as mean ± standard deviation or median (25^th^ and 75^th^), whereas those of the qualitative variables were presented as percentages. Chi-square test, t test, and Wilcoxon test were used as appropriate. All statistical tests were two-sided, and a *P* value < 0.05 was considered statistically significant.

## Results

### Characteristics of participating hospitals

Of the 382 hospitals invited, 222 (58.1% response rate) accepted and completed the online questionnaire. No incomplete datasets were received. There were 125 (56.3%) secondary care hospitals and 97 (43.7%) tertiary care hospitals. Participants were classified into four categories based on the number of hospital beds: 100–350 beds (85), 351–700 beds (65), 701–1200 (45), and more than 1200 beds (27). The majority of hospitals (n = 211) had an independent IPC department, whereas 11 hospitals had IPC department structured within the nursing department or hospital administration departments. For more than 15 years, 133 (63.0%) of the 211 hospitals have an established IPC department (Table 1). In this study, the majority of IPs were women (90.1%), with an average age of 43.4 ± 8.9 years old. The average number of IPs per 100 beds was 0.5. There was no significant difference in the number of IPs per 100 beds between tertiary and secondary care hospitals (*P* = 0.464). The detailed results of the survey are given in Table 2.

**Table 1:** General characteristics of 222 participating hospitals in the study

	Number (%)	IPCAF score Median (IQR)
Hospital type		
Secondary care	125 (56.3)	655 (600–692.5)
Tertiary care	97 (43.7)	720 (682–740)
Region		
Hangzhou	34 (15.3)	686 (615–730.5)
Ningbo	38 (17.1)	682.5 (642.5–718)
Wenzhou	19 (8.6)	632.5 (592.5–720)
Shaoxing	21 (9.5)	705 (632.5–722.5)
Huzhou	13 (5.9)	692.5 (636–731)
Jiaxing	16 (7.2)	712.5 (645.5–738)
Jinhua	27 (12.2)	681 (605–737.5)
Quzhou	10 (4.5)	695 (687.5–731)
Taizhou	19 (8.6)	657.5 (600–727.5)
Lishui	17 (7.7)	657.5 (624–681)
Zhoushan	8 (3.6)	650 (549–686)
Hospital size		
100–350 beds	85 (38.3)	645 (594–686)
351–700 beds	65 (29.3)	681 (619–721)
701–100 beds	31 (14.0)	697.5 (665–735)
≥ 1001 beds	41 (18.5)	730 (710–755)
Independent IPC department		
Yes	211 (95.0)	685 (632.5–725)
No	11 (5.0)	572.5 (532.5–645)
Establishing date of IPC department (n = 211)		
< 5 years	19 (9.0)	642.5 (587.5–692.5)
5–15 years	59 (28.0)	665 (607.5–705)
16–30 years	123 (58.3)	697.5 (657.5–730)
≥ 31 years	10 (4.7)	720 (650–737)
Number of infection preventionists		
0–2	112 (50.5)	645 (587.5–684.5)
3–5	86 (38.7)	710 (667–732.5)
≥ 6	24 (10.8)	734 (720–757)

*IPCAF* infection prevention and control assessment framework, *IPC* Infection prevention and control, *IQR* interquartile range

**Table 2:** Characteristics of infection preventionists in the study

	Total (n = 657)	Tertiary care (n = 415)	Secondary care (n = 242)	*P* value^a^
Age (years)				0.04^b^
20–30	59 (9.0)	39 (9.4)	20 (8.3)	
31–40	189 (28.8)	131 (31.6)	58 (24.0)	
41–50	256(39.0)	145 (34.9)	111 (45.9)	
> 50	153 (23.3)	100 (24.1)	53 (21.9)	
Gender				< 0.001^b^
Male	65 (9.9)	55 (13.3)	10 (4.1)	
Female	592 (90.1)	360 (86.7)	232 (95.9)	
Education level				< 0.001^b^
Junior college and below	72 (11.0)	38 (15.7)	34 (14.0)	
Undergraduate	523 (79.6)	316 (76.1)	207 (85.5)	
Postgraduate and above	62 (9.4)	61 (25.2)	1 (0.4)	
Background				< 0.001^b^
Nursing	478 (72.8)	261 (62.9)	217 (89.7)	
Public health	59 (9.0)	54 (13.0)	5 (2.1)	
Internal medicine	85 (12.9)	73 (17.6)	12 (5.0)	
Laboratory medicine	19 (2.9)	13 (3.1)	6 (2.5)	
Other	16 (2.4)	14 (3.4)	2 (0.8)	
Number of infection preventionists/100 beds	0.5	0.4	0.5	0.464^c^

^a^Comparing tertiary care hospitals and secondary care hospitals

^b^Chi-square test

^c^T test

### The distribution of IPCAF scores

**Fig. 1:**
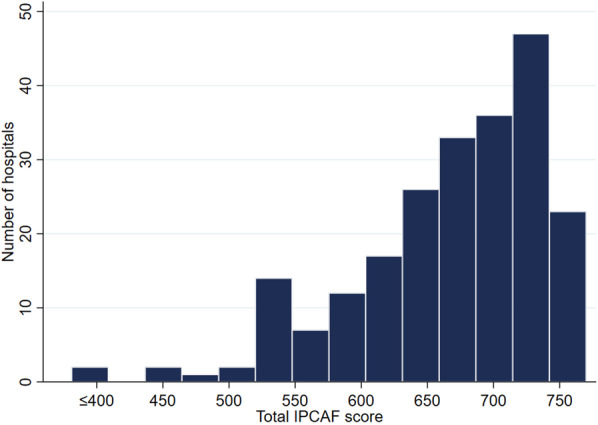
The distribution of IPCAF scores among participating hospitals

**Fig. 2:**
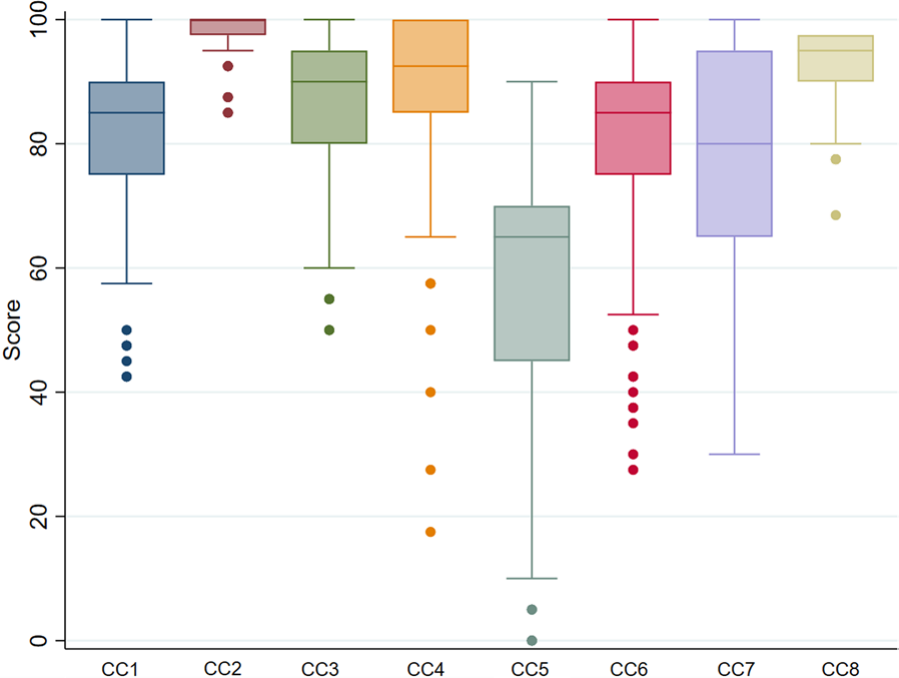
The distribution of scores of the eight core components. The maximum score for each CC was 100. Box plot showing the median, inter-quartile range and outliers of CC

The overall median score of the participating hospitals who were participated was 682 (630–723), with tertiary care hospitals and secondary care hospitals scoring 720 (682–740) and 655 (600–692.5), respectively. There was a significant difference in scores between hospital types (*P* < 0.001). The IPCAF scores were positively correlated with the number of beds and the number of IPs in this study. Figure 1 illustrates the distribution of IPCAF scores among participating hospitals. According to the IPC level assigned by WHO, 184 (82.9%) hospitals achieved an “advanced” IPC level, 36 (16.2%) hospitals reached an “intermediate” level, and 2 (0.9%) hospitals achieved a “basic” level. None of the participating hospitals qualified as “inadequate”. Figure 2 depicts the distribution of scores of individual components, with CC2 (IPC guidelines) and CC6 (Monitoring/audit of IPC practices and feedback) having the highest (100) and lowest (65) median scores, respectively.

### IPC programme

Nearly all hospitals had an IPC committee (96.8%) and IPC objectives (98.2%). A more detailed question about the IPC objectives revealed that approximately half of the hospitals (n = 113) had measurable outcome indicators and set future targets, whereas 35 hospitals (15.8%) had measurable outcome indicators without set future targets. According to the survey, 79.7% of the hospitals (n = 177) had an IPC budget.

### IPC education and training

155 (69.8%) hospitals had additional non-IPC personnel with adequate skills to serve as trainers and mentors. About half (n = 114) of the hospitals stated that they use interactive training sessions, in addition to traditional methods, to train health care workers. To minimize the risk of HAIs, 73.4% of hospitals reported conducting specific IPC training for patients or family members.

### HAI surveillance

Nearly all hospitals (97.3%) reported having personnel responsible for surveillance activities. Only 46 (20.7%) hospitals reported lack of IT support to conduct HAI surveillance. Surveillance of multidrug-resistant (MDR) pathogens according to the local epidemiology situation was reportedly conducted in 212 (95.5%) participating hospitals. 87.8% of hospitals (n = 195) had microbiological laboratory capacity to support MDR surveillance. More than 85% of hospitals had personnel responsible for analyzing and reporting MDR patterns on a regular basis.

### Multimodal strategies for implementation of IPC interventions

The survey indicated that 85.6% of participating hospitals (n = 190) used multimodal strategies to implement IPC interventions, with multidisciplinary teams involved in 164 (73.9%) hospitals. However, 67 (30.2%) hospitals reported not including cultural change in multimodal strategies.

### IPC monitoring and audit

According to the survey, nearly all hospitals (99.1%) had trained personnel responsible for monitoring/audit of IPC practices. However, different hospitals covered different monitoring processes. The survey indicated that 118 (53.2%) hospitals monitored wound dressing change, while 163 (73.4%) hospitals reported antimicrobial agent consumption. 213 (95.9%) participating hospitals reported conducting hand hygiene compliance monitoring, while only 47.7% of hospitals used the WHO hand hygiene survey on a regular basis. Almost all hospitals (n = 219) indicated that they reported monitoring data at least annually. The majority of hospitals (78.8%) performed in a “blame-free” institutional culture for improving IPC practices, whereas only 23 (10.4%) hospitals reported assessing safety culture.

### Workload, staffing and bed occupancy

Most participating hospitals (n = 190) reported having an assessment of staffing needs using national or international standards. 77% of hospitals reported that no extra beds were placed in the corridor. Nevertheless, less than half of the hospitals had adequate spacing of > 1 m between patient beds in all departments. Detailed survey results are presented in the online supplement of this article (Additional file 1).

## Discussion

This survey provided a comprehensive analysis of IPC implementation in Eastern China. Overall, the participating hospitals had a median score of 682, which corresponded to an “advanced” IPC level. These results are similar to those reported in developed countries, such as Germany and Austria. Noticeably, 25.6% (32/125) of the secondary care hospitals that participated were classified as “intermediate” or “basic”. Secondary care hospitals in IPC have limited human and material resources, making some core components difficult to implement. Our results are consistent with the aforementioned phenomenon. Therefore, we should pay more attention to IPC practices in secondary care hospitals, especially IPC programme and multimodal strategies for implementation of IPC interventions.

Nosocomial infection management has been practiced in China since 1896. Over the last 30 years, IPC has made remarkable achievements in regulations and policies, particularly in the aftermath of SARS and COVID-19 pandemics [15, 16]. A median score of 85 to 100 indicated that IPC programs and guidelines are widely established in China. In this study, 96.8% of hospitals reported having an IPC committee, and three-quarters had an allocated budget, both of which were slightly higher than previously reported levels in a systematic review [17]. This is partially explained by the widespread use of electronic HAI surveillance and IPC activities in the participating hospitals. However, further research is required to evaluate the impact of the allocated budget on IPC implementation. There are many national IPC documents, most of which are mandatory in China. Thus, it is not surprising that participating hospitals had such high scores on CC2 (IPC guidelines).

The authors of a 2017 systematic review and expert consensus recommended a ratio of one full-time or equivalent IP per 250 beds [5]. During the COVID-19 pandemic, the National Health Commission of China recommended a ratio of one full-time IP for every 150–200 beds, two IPs for less than 100 beds, and four IPs for 100–500 beds [18]. In our study, the average number of IPs per 100 beds was 0.5, which is lower than 1.2 in American hospitals and 0.8 in Canadian hospitals [19, 20]. There is still a significant disparity between the current staffing levels and national standards, which may be attributed to a lack of commitment by hospital leaders to infection control. Moreover, health care workers may be reluctant to work full-time on IPC due to limited career tracks and low salaries.

Infection control is a dynamic and evolving discipline. Orientation of new employees and continuous IPC education and training for healthcare workers (HCWs) are vital in hospitals. Although HCWs are aware of the risk of the transmission of infection, compliance with standard precautions was unsatisfactory [21]. Therefore, a multifaceted approach to educate HCWs regarding compliance with standard precautions is recommended. Only about half of the participating hospitals reported using simulation-based training, an evidence-based approach that has been shown to improve hand hygiene compliance and lower HAIs, to educate their HCWs and other personnel. Additionally, our results indicated that patients and family members received less IPC training than HCWs. To prevent the transmission of HAIs and improve care quality, recommendations such as hand hygiene, couth etiquette and other IPC that can be shared with the patients to minimize the risk of HAIs were made to empower patients and family members in infection control aspects [22, 23]. Additional studies are needed to assess patient education on infection control measures to develop a validated intervention program.

Surveillance and feedback of surveillance data to frontline HCWs and other stakeholders have been a cornerstone in IPC program improvement. Almost all hospitals reported conducting HAI surveillance, because annual prevalence investigations on HAIs are mandatory in Mainland China. In 2017, the CHINET surveillance reported that the prevalence of methicillin-resistant *S. aureus* was 37.3%, and the prevalence of vancomycin-resistant enterococci was 2.0% [24]. These numbers are alarming, and IPC should be empowered to prevent cross-transmission of multidrug-resistant microorganisms in hospitals.

Assessment of safety culture is still in its infancy among acute care hospitals in China. In our study, only 10.4% of the participating hospitals assessed safety cultural factors. Although the importance of adhering to IPC recommendations has been highlighted, adoption remains suboptimal in hospitals [25]. Safety culture is recognized as a critical factor in improving IPC performance and reducing HAIs [26, 27]. It is recommended that hospitals and care units should regularly assess safety culture using the validated tools and identify areas for improvement.

Our study had certain limitations. The hospitals participated in this study were secondary and tertiary care hospitals, which have more resources for IPC implementation than primary care hospitals. Thus, the IPCAF results can only be extrapolated cautiously to secondary or above care hospitals, but cannot represent the situation of Chinese acute care facilities as a whole. Although there are many Chinese footnotes and explanations provided, some terminology of IPCAF may difficult to understand. The respondents have different educational level, background and work experience, which may lead to false reporting. Furthermore, with a response rate of 58.1%, reporting bias may be present because the results were dependent on hospitals that were interested in participating in this survey. Since this study was conducted during the COVID-19 pandemic, the relatively low response rate may be due to the busy work of IPs and unwillingness to share deficiencies. Notwithstanding these limitations, this study had numerous strengths. This is the first study to use IPCAF to evaluate the current situation of IPC resources and practices in China, which contributes to understand the gap between domestic and international IPC standards. The results highlighted that the IPCAF scores were positively correlated with hospital levels, the number of hospital beds and the number of IPs.

## Conclusion

Overall, IPC structures are at a relatively high level in acute care hospitals in China. The identified potential areas for improvement were similar to those identified in developed countries, particularly regarding multimodal strategies for implementation and safety culture construction. Meanwhile, the Chinese government should pay more attention to IPC resources and practices among secondary care hospitals.

## Supplementary Information

**Additional file 1**. Results of the Infection Prevention and Control Assessment Framework in 222 participating hospitals.

## Data Availability

The datasets in the study are available from the corresponding author on reasonable request.

## Abbreviations

HAIs: Healthcare-associated infections
WHO: World health organization
IPC: Infection prevention and control
COVID-19: Coronavirus disease 2019
IPCAF: Infection prevention and control assessment framework
CC: Core component
NICQI: Nosocomial infection control and quality improvement
HCW: Healthcare worker
