# Supplementary material for: The status of infection prevention and control structures in Eastern China based on the IPCAF tool of the World Health Organization
Source: Antimicrob Resist Infect Control. 2022 Mar 9;11:46. doi: 10.1186/s13756-022-01087-x (PMC8905555; doi:10.1186/s13756-022-01087-x)
Supplement: Supplementary file 1 — Additional file 1. Results of the Infection Prevention and Control Assessment Framework in 222 participating hospitals. [file 13756_2022_1087_MOESM1_ESM.docx]

**Additional File 1: Results of the Infection Prevention and Control Assessment Framework in 222 participating hospitals**

**Core component 1:** **Infection Prevention and Control (IPC) programme**

| **Question/Answer** | **Number (%)** |
| --- | --- |
| **1. Do you have an IPC programme?**  No  Yes, without clearly defined objectives  Yes, with clearly defined objectives and annual activity plan  **2. Is the IPC programme supported by an IPC team comprising of IPC professionals?**  No  Not a team, only an IPC focal person  Yes  **3. Does the IPC team have at least one full-time IPC professional or equivalent (nurse or doctor working 100% in IPC) available?**  No IPC professional available  No, only a part-time IPC professional available  Yes, one per > 250 beds  Yes, one per ≤ 250 beds  **4. Does the IPC team or focal person have dedicated time for IPC activities?**  No  Yes  **5. Does the IPC team include both doctors and nurses?**  No  Yes  **6. Do you have an IPC committee actively supporting the IPC team?**  No  Yes  **7. Are any of the following professional groups represented/included in the IPC committee?**  **- Senior facility leadership (for example, administrative director, chief executive officer [CEO], medical director)**  No  Yes  **- Senior clinical staff (for example, physician, nurse)**  No  Yes  **- Facility management (for example, biosafety, waste, and those tasked with addressing water, sanitation, and hygiene [WASH])**  No  Yes  **8. Do you have clearly defined IPC objectives (that is, in specific critical areas)?**  No  Yes, IPC objectives only  Yes, IPC objectives and measurable outcome indicators (that is, adequate measures for improvement)  Yes, IPC objectives, measurable outcome indicators and set future targets  **9. Does the senior facility leadership show clear commitment and support for the IPC programme:**  **- By an allocated budget specifically for the IPC programme (that is, covering IPC activities, including salaries)?**  No  Yes  **- By demonstrable support for IPC objectives and indicators within the facility (for example, at executive level meetings, executive rounds, participation in morbidity and mortality meetings)?**  No  Yes  **10. Does your facility have microbiological laboratory support (either present on or off site) for routine day-to-day use?**  No  Yes, but not delivering results reliably (timely and of sufficient quality)  Yes, and delivering results reliably (timely and of sufficient quality) | 1 (0.5)  26 (11.7)  195 (87.8)  2 (0.9)  79 (35.6)  141 (63.5)  15 (6.8)  1 (0.5)  59 (26.6)  147 (66.2)  8 (3.6)  214 (96.4)  141 (63.5)  81 (36.5)  7 (3.2)  215 (96.8)  14 (6.3)  208 (93.7)  14 (6.3)  208 (93.7)  14 (6.3)  208 (93.7)  4 (1.8)  70 (31.5)  35 (15.8)  113 (50.9)  45 (20.3)  177 (79.7)  14 (6.3)  208 (93.7)  7 (3.2)  8 (3.6)  207 (93.2) |

**Core component 2:** **Infection Prevention and Control (IPC) guidelines**

| **Question** | **Number (%)** |
| --- | --- |
| **1. Does your facility have the expertise (in IPC and/or infectious diseases) for developing or adapting guidelines?**  No  Yes  **2. Does your facility have guidelines available for:**  **- Standard precautions?**  No  Yes  **- Hand hygiene?**  No  Yes  **- Transmission-based precautions?**  No  Yes  **- Outbreak management and preparedness?**  No  Yes  **- Prevention of surgical site infection?**  No  Yes  **- Prevention of vascular catheter-associated bloodstream infections?**  No  Yes  **- Prevention of hospital-acquired pneumonia ([HAP]; all types of HAP, including (but not exclusively) ventilator-associated pneumonia)?**  No  Yes  **- Prevention of catheter-associated urinary tract infections?**  No  Yes  **- Prevention of transmission of multidrug-resistant (MDR) pathogens?**  No  Yes  **- Disinfection and sterilization?**  No  Yes  **- Health care worker protection and safety**  No  Yes  **- Injection safety?**  No  Yes  **- Waste management?**  No  Yes  **- Antibiotic stewardship?**  No  Yes  **3. Are the guidelines in your facility consistent with national/international guidelines (if they exist)?**  No  Yes  **4. Is implementation of the guidelines adapted according to the local needs and resources while maintaining key IPC standards?**  No  Yes  **5. Are frontline health care workers involved in both planning and executing the implementation of IPC guidelines in addition to IPC personnel?**  No  Yes  **6. Are relevant stakeholders (for example, lead doctors and nurses, hospital managers, quality management) involved in the development and adaptation of the IPC guidelines in addition to IPC personnel?**  No  Yes  **7. Do health care workers receive specific training related to new or updated IPC guidelines introduced in the facility?**  No  Yes  **8. Do you regularly monitor the implementation of at least some of the IPC guidelines in your facility?**  No  Yes | 0 (0.0)  222 (100.0)  0 (0.0)  222 (100.0)  0 (0.0)  222 (100.0)  0 (0.0)  222 (100.0)  11 (5.0)  211 (95.0)  0 (0.0)  222 (100.0)  63 (28.4)  159 (71.6)  49 (22.1)  173 (77.9)  0 (0.0)  222 (100.0)  0 (0.0)  222 (100.0)  0 (0.0)  222 (100.0)  0 (0.0)  222 (100.0)  0 (0.0)  222 (100.0)  0 (0.0)  222 (100.0)  0 (0.0)  222 (100.0)  0 (0.0)  222 (100.0)  0 (0.0)  222 (100.0)  0 (0.0)  222 (100.0)  4 (1.8)  218 (98.2)  0 (0.0)  222 (100.0)  0 (0.0)  222 (100.0) |

**Core component 3:** **Infection Prevention and Control (IPC) education and training**

| **Question** | **Number (%)** |
| --- | --- |
| **1. Are there personnel with the IPC expertise (in IPC and/or infectious diseases) to lead IPC training?**  No  Yes  **2. Are there additional non-IPC personnel with adequate skills to serve as trainers and mentors (for example, link nurses or doctors, champions)?**  No  Yes  **3. How frequently do health care workers receive training regarding IPC in your facility?**  Never or rarely  New employee orientation only for health care workers  New employee orientation and regular (at least annually) IPC training for health care workers offered but not mandatory  New employee orientation and regular (at least annually) mandatory IPC training for all health care workers  **4. How frequently do cleaners and other personnel directly involved in patient care receive training regarding IPC in your facility?**  Never or rarely  New employee orientation only for other personnel  New employee orientation and regular (at least annually) training for other personnel offered but not mandatory  New employee orientation and regular (at least annually) mandatory IPC training for other personnel  **5. Does administrative and managerial staff receive general training regarding IPC in your facility?**  No  Yes  **6. How are health care workers and other personnel trained?**  No training available  Using written information and/or oral instruction and/or e-learning only  Includes additional interactive training sessions (for example, simulation and/or bedside training)  **7. Are there periodic evaluations of the effectiveness of training programmes (for example, hand hygiene audits, other checks on knowledge)?**  No  Yes, but not regularly  Yes, regularly (at least annually)  **8. Is IPC training integrated in the clinical practice and training of other specialties (for example, training of surgeons involves aspects of IPC)?**  No  Yes, in some disciplines  Yes, in all disciplines  **9. Is there specific IPC training for patients or family members to minimize the potential for health care-associated infections (for example, immunosuppressed patients, patients with invasive devices, patients with multidrug-resistant infections)?**  No  Yes  **10. Is ongoing development/education offered for IPC staff (for example, by regularly attending conferences, courses)?**  No  Yes | 3 (1.4)  219 (98.6)  67 (30.2)  155 (69.8)  0 (0)  0 (0)  29 (13.1)  193 (86.9）  0 (0)  1 (0.5)  32 (14.4)  189 (85.1)  27 (12.2)  195 (87.8)  0 (0)  108 (48.6)  114 (51.4)  0 (0)  54 (24.3)  168 (75.7)  33 (14.9)  119 (53.6)  70 (31.5)  59 (26.6)  163 (73.4)  1 (0.5)  221 (99.5) |

**Core component 4: Health care-associated infection (HAI) surveillance**

| **Question** | **Number (%)** |
| --- | --- |
| **1. Is surveillance a defined component of your IPC programme?**  No  Yes  **2. Do you have personnel responsible for surveillance activities?**  No  Yes  **3. Have the professionals responsible for surveillance activities been trained in basic epidemiology, surveillance and IPC (that is, capacity to oversee surveillance methods, data management and interpretation)?**  No  Yes  **4. Do you have informatics/IT support to conduct your surveillance (for example, equipment, mobile technologies, electronic health records)?**  No  Yes  **5. Do you go through a prioritization exercise to determine the HAIs to be targeted for surveillance according to the local context (that is, identifying infections that are major causes of morbidity and mortality in the facility)?**  No  Yes  **6. In your facility is surveillance conducted for:**  **- Surgical site infections?**  No  Yes  **- Device-associated infections (for example, catheter-associated urinary tract infections, central line-associated bloodstream infections, peripheral-line associated bloodstream infections, ventilator-associated pneumonia)?**  No  Yes  **- Clinically-defined infections (for example, definitions based only on clinical signs or symptoms in the absence of microbiological testing)?**  No  Yes  **- Colonization or infections caused by multidrug-resistant pathogens according to your local epidemiological situation?**  No  Yes  **- Local priority epidemic-prone infections (for example, norovirus, influenza, tuberculosis [TB], severe acute respiratory syndrome [SARS], Ebola, Lassa fever)?**  No  Yes  **- Infections in vulnerable populations (for example, neonates, intensive care unit, immunocompromised, burn patients)?**  No  Yes  **- Infections that may affect health care workers in clinical, laboratory, or other settings (for example, hepatitis B or C, human immunodeficiency virus [HIV], influenza)?**  No  Yes  **7. Do you regularly evaluate if your surveillance is in line with the current needs and priorities of your facility?**  No  Yes  **8. Do you use reliable surveillance case definitions (defined numerator and denominator according to international definitions [e.g. CDC NHSN/ECDC] or if adapted, through an evidence-based adaptation process and expert consultation?**  No  Yes  **9. Do you use standardized data collection methods (for example, active prospective surveillance) according to international surveillance protocols (for example, CDC NHSN/ECDC) or if adapted, through an evidence-based adaptation process and expert consultation?**  No  Yes  **10. Do you have processes in place to regularly review data quality (for example, assessment of case report forms, review of microbiology results, denominator determination, etc.)?**  No  Yes  **11. Do you have adequate microbiology and laboratory capacity to support surveillance?**  No  Yes, can differentiate gram-positive/negative strains but cannot identify pathogens  Yes, can reliably identify pathogens (for example, isolate identification) in a timely manner  Yes, can reliably identify pathogens and antimicrobial drug resistance patterns (that is, susceptibilities) in a timely manner  **12. Are surveillance data used to make tailored unit/facility-based plans for the improvement of IPC practices?**  No  Yes  **13. Do you analyze antimicrobial drug resistance on a regular basis (for example, quarterly/half-yearly/annually)?**  No  Yes  **14. Do you regularly (for example, quarterly/half-yearly/annually) feedback up-to-date surveillance information to:**  **- Frontline health care workers (doctors/nurses)?**  No  Yes  **- Clinical leaders/heads of department**  No  Yes  **- IPC committee**  No  Yes  **- Non-clinical management/administration (chief executive officer/chief financial officer)?**  No  Yes  **15. How do you feedback up-to-date surveillance information? (at least annually)**  No feedback  By written/oral information only  By presentation and interactive problem-orientated solution finding | 2 (0.9)  220 (99.1)  6 (2.7)  216 (97.3)  35 (15.8)  187 (84.2)  46 (20.7)  176 (79.3)  7 (3.2)  215 (96.8)  22 (9.9)  200 (90.1)  19 (8.6)  203 (91.4)  25 (11.3)  197 (88.7)  10 (4.5)  212 (95.5)  83 (37.4)  139 (62.6)  51 (23.0)  171 (77.0)  7 (3.2)  215 (96.8)  45 (20.3)  177 (79.7)  0 (0.0)  222 (100.0)  8 (3.6)  214 (96.4)  2 (0.9)  220 (99.1)  7 (3.2)  6 (2.7)  14 (6.3)  195 (87.8)  21 (9.5)  201 (90.5)  32 (14.4)  190 (85.6)  10 (4.5)  212 (95.5)  4 (1.8)  218 (98.2)  5 (2.3)  217 (97.7)  5 (2.3)  217 (97.7)  0 (0)  96 (43.2)  126 (56.8) |

**Core component 5: Multimodal strategies for implementation of infection prevention and control (IPC) interventions**

| **Question** | **Number (%)** |
| --- | --- |
| **1. Do you use multimodal strategies to implement IPC interventions?**  No  Yes  **2. Do your multimodal strategies include any or all of the following elements:**  **- System change**  Element not included in multimodal strategies  Interventions to ensure the necessary infrastructure and continuous availability of supplies are in place  Interventions to ensure the necessary infrastructure and continuous availability of supplies are in place and addressing ergonomics and accessibility, such as the best placement of central venous catheter set and tray  **- Education and training**  Element not included in multimodal strategies  Written information and/or oral instruction and/or e-learning only  Additional interactive training sessions (includes simulation and/or bedside training)  **- Monitoring and feedback**  Element not included in multimodal strategies  Monitoring compliance with process or outcome indicators (for example, audits of hand hygiene or catheter practices)  Monitoring compliance and providing timely feedback of monitoring results to health care workers and key players  **- Communications and reminders**  Element not included in multimodal strategies  Reminders, posters, or other advocacy/awareness-raising tools to promote the intervention  Additional methods/initiatives to improve team communication across units and disciplines (for example, by establishing regular case conferences and feedback rounds)  **- Safety climate and culture change**  Element not included in multimodal strategies  Managers/leaders show visible support and act as champions and role models, promoting an adaptive approach and strengthening a culture that supports IPC, patient safety and quality  Additionally, teams and individuals are empowered so that they perceive ownership of the intervention (for example, by participatory feedback rounds)  **3. Is a multidisciplinary team used to implement IPC multimodal strategies?**  No  Yes  **4. Do you regularly link to colleagues from quality improvement and patient safety to develop and promote IPC multimodal strategies?**  No  Yes  **5. Do these strategies include bundles or checklists?**  No  Yes | 32 (14.4)  190 (85.6)  19 (8.6)  193 (86.9)  10 (4.5)  5 (2.3)  202 (91.0)  15 (6.8)  10 (4.5)  65 (29.3)  147 (66.2)  23 (10.4)  149 (67.1)  50 (22.5)  67 (30.2)  114 (51.4)  41 (18.5)  66 (29.7)  156 (70.3)  58 (26.1)  164 (73.9)  47 (21.2)  175 (78.8) |

**Core component 6: Monitoring/audit of IPC practices and feedback**

| **Question** | **Number (%)** |
| --- | --- |
| **1. Do you have trained personnel responsible for monitoring/audit of IPC practices and feedback?**  No  Yes  **2. Do you have a well-defined monitoring plan with clear goals, targets and activities (including tools to collect data in a systematic way)?**  No  Yes  **3. Which processes and indicators do you monitor in your facility?** (Tick all that apply)  None  Hand hygiene compliance (using the WHO hand hygiene observation tool or equivalent)  Intravascular catheter insertion and/or care  Wound dressing change  Transmission-based precautions and isolation to prevent the spread of multidrug resistant organisms (MDRO)  Cleaning of the ward environment  Disinfection and sterilization of medical equipment/instruments  Consumption/usage of alcohol-based handrub or soap  Consumption/usage of antimicrobial agents  Waste management  **4. How frequently is the WHO Hand Hygiene Self-Assessment Framework Survey undertaken?**  Never  Periodically, but no regular schedule  At least annually  **5. Do you feedback auditing reports (for example, feedback on hand hygiene compliance data or other processes) on the state of the IPC activities/performance?** (Tick all that apply)  No reporting  Yes, within the IPC team  Yes, to department leaders and managers in the areas being audited  Yes, to frontline health care workers  Yes, to the IPC committee or quality of care committees or equivalent  Yes, to hospital management and senior administration  **6. Is the reporting of monitoring data undertaken regularly (at least annually)?**  No  Yes  **7. Are monitoring and feedback of IPC processes and indicators performed in a “blame-free” institutional culture aimed at improvement and behavioural change?**  No  Yes  **8. Do you assess safety cultural factors in your facility (for example, by using other surveys such as HSOPSC, SAQ, PSCHO, HSC)**  No  Yes | 2 (0.9)  220 (99.1)  13 (5.9)  209 (94.1)  0 (0.0)  213 (95.9)  170 (76.6)  118 (53.2)  188 (84.7)  200 (90.1)  199 (89.6)  189 (85.1)  163 (73.4)  200 (90.1)  58 (26.1)  58 (26.1)  106 (47.7)  0 (0.0)  160 (72.1)  202 (91.0)  182 (82.0)  190 (85.6)  164 (73.9)  3 (1.4)  219 (98.6)  47 (21.2)  175 (78.8)  199 (89.6)  23 (10.4) |

**Core component 7: Workload, staffing and bed occupancy**

| **Question** | **Number (%)** |
| --- | --- |
| **1. Are appropriate staffing levels assessed in your facility according to patient workload using national standards or a standard staffing needs assessment tool such as the WHO Workload indicators of staffing need method?**  No  Yes  **2. Is an agreed (that is, WHO or national) ratio of health care workers to patients maintained across your facility?**  No  Yes, for staff in less than 50% of units  Yes, for staff in more than 50% of units  Yes, for all health care workers in the facility  **3. Is a system in place in your facility to act on the results of the staffing needs assessments when staffing levels are deemed to be too low?**  No  Yes  **4. Is the design of wards in your facility in accordance with international standards regarding bed capacity?**  No  Yes, but only in certain departments  Yes, for all departments (including emergency department and pediatrics)  **5. Is bed occupancy in your facility kept to one patient per bed?**  No  Yes, but only in certain departments  Yes, for all units (including emergency departments and pediatrics)  **6. Are patients in your facility placed in beds standing in the corridor outside of the room (including beds in the emergency department)?**  Yes, more frequently than twice a week  Yes, less frequently than twice a week  No  **7. Is adequate spacing of > 1 meter between patient beds ensured in your facility?**  No  Yes, but only in certain departments  Yes, for all departments (including emergency department and pediatrics)  **8. Is a system in place in your facility to assess and respond when adequate bed capacity is exceeded?**  No  Yes, this is the responsibility of the head of department  Yes, this is the responsibility of the hospital administration/management | 32 (14.4)  190 (85.6)  18 (8.1)  37 (16.7)  138 (62.2)  29 (13.1)  24 (10.8)  198 (89.2)  12 (5.4)  74 (33.3)  136 (61.3)  0 (0.0)  0 (0.0)  222 (100.0)  22 (9.9)  29 (13.1)  171 (77.0)  20 (9.0)  94 (42.3)  108 (48.6)  15 (6.8)  57 (25.7)  150 (67.6) |

**Core component 8: Built environment, materials and equipment for IPC at the facility level**

| **Question** | **Number (%)** |
| --- | --- |
| **1. Are water services available at all times and of sufficient quantity for all uses (for example, hand washing, drinking, personal hygiene, medical activities, sterilization, decontamination, cleaning and laundry)?**  No, available on average < 5 days per week  Yes, available on average ≥ 5 days per week or every day but not of sufficient quantity  Yes, every day and of sufficient quantity  **2. Is a reliable safe drinking water station present and accessible for staff, patients and families at all times and in all locations/wards?**  No, not available  Sometimes, or only in some places or not available for all users  Yes, accessible at all times and for all wards/groups  **3. Are functioning hand hygiene stations (that is, alcohol-based handrub solution or soap and water and clean single-use towels) available at all points of care?**  No, not present  Yes, stations present, but supplies are not reliably available  Yes, with reliably available supplies  **4. In your facility, are ≥ 4 toilets or improved latrines available for outpatient settings or ≥ 1 per 20 users for inpatient settings?**  Less than required number of toilets or latrines available and functioning  Sufficient number present but not all functioning  Sufficient number present and functioning  **5. In your health care facility, is sufficient energy/power supply available at day and night for all uses (for example, pumping and boiling water, sterilization and decontamination, incineration or alternative treatment technologies, electronic medical devices, general lighting of areas where health care procedures are performed to ensure safe provision of health care and lighting of toilet facilities and showers)?**  No  Yes, sometimes or only in some of the mentioned areas  Yes, always and in all mentioned areas  **6. Is functioning environmental ventilation (natural or mechanical) available in patient care areas?**  No  Yes  **7. For floors and horizontal work surfaces, is there an accessible record of cleaning, signed by the cleaners each day?**  No record of floors and surfaces being cleaned  Record exists, but is not completed and signed daily or is outdated  Yes, record completed and signed daily  **8. Are appropriate and well-maintained materials for cleaning (for example, detergent, mops, buckets, etc.) available?**  No materials available  Yes, available but not well maintained  Yes, available and well-maintained  **9. Do you have single patient rooms or rooms for cohorting patients with similar pathogens if the number of isolation rooms is insufficient (for example, TB, measles, cholera, Ebola, SARS)?**  No  No single rooms but rather rooms suitable for patient cohorting available  Yes, single rooms are available  **10. Is PPE available at all times and in sufficient quantity for all uses for all health care workers?**  No  Yes, but not continuously available in sufficient quantities  Yes, continuously available in sufficient quantities  **11. Do you have functional waste collection containers for non-infectious (general) waste, infectious waste and, sharps waste in close proximity to all waste generation points?**  No bins or separate sharps disposal  Separate bins present but lids missing or more than 3/4 full; only two bins (instead of three); or bins at some but not all waste generation points  Yes  **12. Is a functional burial pit/fenced waste dump or municipal pick-up available for disposal of non-infectious (non-hazardous/general waste)?**  No pit or other disposal method used  Pit in facility but insufficient dimensions; pits/dumps overfilled or not fenced/locked; or irregular municipal waste pick up  Yes  **13. Is an incinerator or alternative treatment technology for the treatment of infectious and sharp waste (for example, an autoclave) present (either present on or off site and operated by a licensed waste management service), functional and of a sufficient capacity?**  No, none present  Present, but not functional  Yes  **14. Is a wastewater treatment system (for example, septic tank followed by drainage pit) present (either on or off site) and functioning reliably?**  No, not present  Yes, but not functioning reliably  Yes and functioning reliably  **15. Does your health care facility provide a dedicated decontamination area and/or sterile supply department (either present on or off site and operated by a licensed decontamination management service) for the decontamination and sterilization of medical devices and other items/equipment?**  No, not present  Yes, but not functioning reliably  Yes and functioning reliably  **16. Do you reliably have sterile and disinfected equipment ready for use?**  No, available on average < five days per week  Yes, available on average ≥ five days per week or every day, but not of sufficient quantity  Yes, available every day and of sufficient quantity  **17. Are disposable items available when necessary? (for example, injection safety devices, examination gloves)**  No, not available  Yes, but only sometimes available  Yes, continuously available | 0 (0.0)  2 (0.9)  220 (99.1)  0 (0.0)  20 (9.0)  202 (91.0)  0 (0.0)  9 (4.1)  213 (95.9)  0 (0.0)  0 (0.0)  222 (100.0)  0 (0.0)  8 (3.6)  214 (96.4)  3 (1.4)  219 (98.6)  37 (16.7)  51 (23.0)  134 (60.4)  2 (0.9)  39 (17.6)  181 (81.5)  7 (3.2)  32 (14.4)  183 (82.4)  0 (0.0)  4 (1.8)  218 (98.2)  0 (0.0)  7 (3.2)  215 (96.8)  0 (0.0)  0 (0.0)  222 (100.0)  10 (4.5)  4 (1.8)  208 (93.7)  0 (0.0)  10 (4.5)  212 (95.5)  2 (0.9)  2 (0.9)  218 (98.2)  7 (3.2)  19 (8.6)  196 (88.3)  0 (0.0)  2 (0.9)  220 (99.1) |
